# An Intervention to Improve Uptake of Referrals for Children with Ear Disease or Hearing Loss in Thyolo District, Malawi: Acceptability and Feasibility

**DOI:** 10.3390/ijerph16173144

**Published:** 2019-08-28

**Authors:** Antonia Baum, Wakisa Mulwafu, Mwanaisha Phiri, Sarah Polack, Tess Bright

**Affiliations:** 1 International Centre for Evidence in Disability, London School of Hygiene & Tropical Medicine, London WC1 E7HT, UK; 2 Department of Surgery, College of Medicine, University of Malawi, Blantyre, Malawi; 3 Audiology Department, Queen Elizabeth Central Hospital, Blantyre, Malawi

**Keywords:** hearing loss, Malawi, children, access, feasibility

## Abstract

(1) *Introduction*: Poor uptake of referral for ear and hearing services in Malawi has been demonstrated in previous research. A multi-component educational intervention was developed to address poor uptake. The aim of this study was to explore the feasibility and acceptability of the intervention. In addition, we aimed to provide a case study of an intervention development and feasibility testing process in preparation for a potential randomised trial. (2) *Methods*: The intervention included three components: (i) an information booklet; (ii) personalised counselling by a community health worker and an expert mother; (iii) a text message reminder. To assess feasibility, the counselling and information booklet were provided for caregivers of eligible children (<18 years) at ear and hearing outreach camps in Thyolo. Text message reminders were sent to caregivers after the camps. After 4 weeks, all caregivers were revisited and completed a structured questionnaire and a subset were interviewed in-depth. (3) *Results*: 30 children were recruited, and 53% took up the referral. Interviews found counselling with a booklet was acceptable. It provided motivation, enabled a two-way conversation, and helped dispel fear. It allowed information to be shared with social networks, initiating conversations about raising funds. The text message reminder was reported to be a valued prompt. Challenges to feasibility included low network coverage, and time needed for counselling. Residual barriers included the costs of transportation. The cost was £3.70/camp. (4) *Conclusions*: The study found that counselling with an information booklet was feasible and acceptable. The process of testing the feasibility of the intervention identified some adaptations to the intervention components and delivery which could be implemented before it is tested in a trial. This study highlighted the value of the feasibility testing process.

## 1. Introduction

Worldwide, an estimated 34 million children have disabling hearing loss, the majority (>80%) of whom live in low- and middle-income countries (LMICs) [1]. The World Health Organization (WHO) defines “disabling” hearing loss in children as a pure tone average (across frequencies 0.5, 1, 2, and 4 kilohertz (kHz)) of more than 30 dB in the better hearing ear [1]. The impact of hearing loss can be profound. In addition to experiencing stigma and discrimination, evidence suggests that children with unmanaged hearing loss have lower levels of literacy and poorer educational attainment compared to children with normal hearing [2,3,4,5]. This, in turn, can limit employment opportunities later in life and increase the risk of poverty [6,7]. 

The WHO estimates that 60% of childhood hearing loss can be prevented, with the remaining treatable through effective interventions such as hearing aids and surgery [8]. According to WHO, more than 30% of hearing loss in children is caused by infections such as rubella, measles and chronic ear infections; a further 17% due to complications at birth (low birthweight, hypoxia); and ototoxic medication accounts for 4% [8]. In many LMICs, there is a substantial shortage of human and technical resources needed to provide ear and hearing services [9]. In Malawi, there are three trained Ear, Nose and Throat (ENT) surgeons and three audiologists for a population of >17 million people, and the vast majority of ear and hearing services are located in urban areas (e.g., Queen Elizabeth Central Hospital (QECH) in Blantyre) [10]. To improve access to ear and hearing services for rural, underserved populations, ear and hearing outreach camps are regularly held in rural areas, which provide hearing assessments and ear examination by clinicians (ENT and audiologists) from QECH. Simple treatments are provided in the camps, and those with more complex conditions are referred. However, evidence suggests that referral uptake following the camps is low, which may limit their value. In a previous study, out of 170 children referred at outreach camps to ear and hearing services at QECH, only 3% were found to take up the referral [11]. Qualitative research found that the key barriers to uptake were: fear and uncertainty about the referral hospital, procedural problems within the camps leading to a lack of understanding about the referral, distance to the hospital, low awareness and understanding of hearing loss, and lack of and cost of transport [11]. 

This low referral uptake needs to be addressed urgently in order to maximise the benefit of the outreach camps. However, evidence is lacking as to how best to achieve this [12,13]. In previously published research, we developed an intervention to improve referral uptake, drawing on recognised frameworks for intervention design (Medical Research Council’s (MRC) guidance and the behaviour centred design (BCD approach) [14]. The resulting intervention included (i) an information booklet delivered with counselling by a trained community health worker and an “expert mother” (i.e., mother of a child with hearing loss who had attended QECH for referral previously) at outreach camps and (ii) a text message reminder to attend the appointment. The aim of this study was to explore the feasibility and acceptability of this intervention from the perspective of both recipients and implementers. We also aimed to provide a case study of an intervention development and feasibility testing process, as recommended by the MRC, in preparation for a potential randomised trial.

## 2. Materials and Methods 

The results of the intervention development stages, including the formative research, systematic reviews, development of a theory of change, and intervention design have been described elsewhere [11,12,13]. This study focused on testing the feasibility, acceptability and costs of the intervention.

### 2.1. The Intervention 

The multi-component intervention, developed through an iterative designer-led process was:

- An information booklet delivered with counselling by a trained health surveillance assistant (HSA) and an “expert mother” at the point at which the referral was made (in outreach camps). The booklet had three sections (Appendix A):

1. An illustrated storyline of “The Banda Family” depicting a family going through the process of being referred and attending the referral at QECH;
2. Information on getting to QECH, including photographs of key locations/buildings/roads that caregivers would see on the way to the ENT department;
3. Tailored action planning section—including how to get to the hospital, how much money is needed, and what they need to bring with them. The majority of services are free at the point of care in Malawi. However, hearing aids may incur out of pocket costs. At the time of writing, the audiology services offered a pay-what-you can system.

The counsellors describe each component of the booklet to the caregiver and child, and also tailor their counselling to each child (e.g., based on the type of referral—for hearing aids or surgery). The expert mother shares her experiences of attending QECH and the consequences of not attending.

- A text message reminder is sent two days before the scheduled appointment, followed by a second text message reminder if they do not attend on the scheduled date. The text message is tailored to the individual, and includes the child’s name, appointment date, and a phone number to call in case of questions.

Each component of the intervention was designed to address the particular barriers raised in the formative research.

### 2.2. Pilot Testing the Intervention 

#### 2.2.1. Study Design 

The MRC guidance on designing and evaluating complex interventions recommends that pilot and feasibility studies be conducted prior to implementing a full trial [14]. The terms “feasibility” and “pilot” have not been used consistently in the literature in studies preparing for a full trial, such as this. However, recent work by Eldridge and colleagues has helped to clarify the differences and relationship between the two [15]. Their conceptual framework suggests that “feasibility study” is a broad term that encompasses different types of studies: randomised pilot studies, non-randomised pilot studies, and feasibility studies that are not pilot studies. The authors suggest that a number of studies can be conducted to assess the feasibility of a randomised control trial. We conducted a non-randomised pilot study, in which our intervention was delivered and evaluated without randomisation of the participants. We did not randomise participants, as we wanted to focus on piloting the intervention itself, rather than trial processes which have been tested widely in the Malawian setting [16,17]. Alongside this, we conducted a feasibility study which was not a pilot, to answer questions around the acceptability of the intervention. Both study designs are types of feasibility studies. 

#### 2.2.2. Setting 

The intervention was pilot tested in three ear and hearing outreach camps in Thyolo. These camps are typically attended by the following clinicians from QECH: ENT specialists/ENT clinical officers, audiologists/audiology officers, and nurses. People requiring specialist care are given verbal referrals and a date to attend QECH by ENT doctors. The clinicians attending the camps have typically had several years of clinical experience in ear and hearing care. In terms of education, ENT specialists have undertaken a medical degree and 5 year ENT specialisation; clinical officers receive 18 months of training in ENT; audiologists a 2-year Masters degree (obtained outside Malawi); and audiology officers a 3-year diploma (also obtained outside Malawi). 

#### 2.2.3. Study Sample

The expectation was that three camps would identify approximately 30 eligible families of children (<18 years) with hearing loss who would receive the counselling intervention. This sample size was considered sufficient for pilot testing, based on previous literature [18]. The eligibility criteria for inclusion in the study were children <18 years, referred to QECH for hearing loss requiring hearing aids or surgery, and attending the camps with a caregiver. Those attending without a caregiver were not included due to ethical considerations. 

#### 2.2.4. Data Collection 

We used mixed methods approach to assess feasibility and acceptability. After receiving counselling at the outreach camp, caregivers were interviewed using a structured questionnaire (which included pre-coded and open text responses) by a trained local research assistant (Mwanaisha Phiri). Data were collected on demographic information about the child and family, the results of their ear and hearing screening assessments, and caregiver reflections on the counselling intervention. After 4 weeks, caregivers were revisited in their homes and interviewed again using a structured questionnaire about attendance, reasons for going/not going, and feedback on the intervention. These data were collected electronically on tablets using Open Data Kit (ODK Development Team). 

In addition, at follow-up, qualitative interviews were undertaken with a sub-sample of participants who were selected purposively according to age, gender and whether or not they attended QECH. We aimed to interview 10 caregivers who attended and 10 who did not. Interviews were conducted in the participants’ homes. Topic guides developed for these interviews covered: history of hearing health, previous care seeking, experiences at the outreach camp, decision-making process for attending QECH, experience attending QECH, and general feedback about the intervention. We also conducted qualitative interviews with the intervention implementers (counsellors and clinicians) to explore the feasibility and fidelity of the intervention—whether it was delivered as we intended. These interviews were conducted at QECH or Thyolo District Hospital. Interviews were conducted in Chichewa (the national language) and audio-recorded, and then transcribed and translated into English for analysis. All caregivers spoke Chichewa fluently. 

Data were collected on estimated costs of the intervention, including: costs of developing the booklet, printing costs, costs of training counsellors, costs of sending text messages, and personnel costs to counsel each caregiver.

#### 2.2.5. Analysis 

A descriptive analysis of the quantitative data was undertaken in Stata (version 15.0, StataCorp LLC, College Station, TX, USA). Qualitative data from in-depth interviews were analysed using interpretive phenomenological analysis (IPA). IPA is well suited to analysing a small, fairly homogenous sample because it is able to develop a detailed understanding of how individuals make sense of experiences [19]. A social anthropologist (Antonia Baum) read through each transcript to become familiar with the data [20]. To develop a rigorous, balanced picture, the qualitative study was triangulated by analysing the data with three different aims: (1) to aggregate feedback about the intervention, (2) to gain a deeper understanding of the factors affecting uptake and (3) to develop case studies for each child. The transcripts were coded for themes and patterns manually using an inductive–deductive approach [21] in which codes were identified and categorised thematically by visual arrangement, and themes were continually refined. To ensure the analysis situated the texts in their local setting, close textual analysis [22] was used to build case studies for each child, paying close attention to discursive motifs, the narrative development in each interview, and the socio-economic and cultural context. As the codes emerged, the data were cross-referenced with the case studies in order to find contextual explanations for particular perspectives and behaviours, thereby helping to refine the codes. 

#### 2.2.6. Positionality

Interviews were conducted in Chichewa by a trained female Malawian research assistant who is also an audiologist (Mwanaisha Phiri). Her background as a health professional may have influenced the interview dynamics with the caregivers. The analysis was conducted by Antonia Baum and Tess Bright, who are both female researchers living in the United Kingdom and their interpretation may have been influenced by their background. For example, some of the contextual details may have been overlooked or misinterpreted. To limit this potential bias, the findings were reviewed by two Malawian researchers with experience working in ear and hearing care in rural settings (Wakisa Mulwafu, Mwanaisha Phiri). 

#### 2.2.7. Ethical Considerations

Informed consent was obtained from all participants before the intervention was received. An information sheet was given to the participants, which outlined the purposes of the study. This was also summarised verbally before consent was obtained. Participants agreed to have their photographs taken as part of this research. Ethical approval was obtained from London School of Hygiene & Tropical Medicine ethics board (14433) and the College of Medicine Research Ethics Committee (COMREC) in Malawi (P.09/17/2278).

## 3. Results

### 3.1. Study Sample 

In total, 30 children were recruited to the study from 28 families (two sets of siblings) from three outreach camps in Thyolo, and were interviewed at baseline and four week follow up. Table 1 provides a description of the sample at baseline (at the camps). The mean age of children in the sample was 10.5 years (range 4–16 years) and 57% were female. Nearly three-quarters (*n* = 22) of caregivers had previously sought care for their child’s ear/hearing condition. The majority of these sought care at the district hospital (*n* = 14). Of the caregivers who attended the camp, 13% were not the primary caregiver for the child. In terms of type of referral given, 37% (*n* = 11) were referred for hearing assessment/hearing aids due to suspected permanent hearing loss, and 63% (*n* = 19) for surgery.

Twenty-six caregivers of 23 children also underwent a qualitative interview. In five families, two caregivers were interviewed, one who had been to the camp and one who had attended QECH, to explore different perspectives. Of those interviewed in depth, 11 did and 12 did not attend the referral at QECH. In addition to the caregivers, seven intervention implementers were interviewed (including the expert mother, HSA and clinicians involved in the camps). 

In total, 16 (53%) of the children took up the referral at QECH. The findings relating to the acceptability and feasibility of the intervention are presented below, first in terms of the counselling and booklet component of the intervention, followed by the text message reminder. 

### 3.2. Counselling and Booklet: Acceptability

#### 3.2.1. Counselling

##### Enabling Two-Way Conversation 

Supporting the medical examinations with individual counselling sessions was appreciated and considered successful by all caregivers and implementers. The counselling enabled a two-way conversation in which caregivers could ask questions; this was cited as particularly “encouraging” and several caregivers recalled the experience with enthusiasm. “I loved it”, one caregiver stated, explaining that it gave her “faith”, which made her determined to take up the referral. Several caregivers, including some who were ultimately unable to attend, reported that after the counselling session, they were “certain” that their child would be taken to QECH. Although most thought they had understood the diagnosis and recommendations given by the doctor prior to counselling, the counselling gave them a supportive framework to digest the information and find out more about what treatment would entail. For instance, a mother described the counselling as:

*Wonderful because we were being encouraged. The doctor can explain to you, yes, but you might have questions, and we were able to have a discussion with them.*

*[Caregiver code 316]*

The local expert mother’s testimony of her uptake of referral to QECH also encouraged many of those who heard it, because they could identify with her perspective. An uncle at the camp said her counselling:

*Was helpful since it encouraged us. She told us that she also comes from Thyolo.*

*[Caregiver 101]*

##### Dispelling Misconceptions and Fear

Counselling improved the acceptability of the referral for the majority of caregivers who assumed that uptake would be prohibitively expensive. Further, most caregivers had never been to a large urban hospital and were unfamiliar with the treatments on offer. Counselling appeared to dispel fear or distrust of QECH. One caregiver said about the counselling:

*It was good because they made us free to see that Queens is not a dangerous place, but [a place] where one can get assistance.*

*[Caregiver 104]*

#### 3.2.2. Booklet

##### Motivation from Storyline

Reactions to all sections of the illustrated booklet were unanimously favourable. The majority of caregivers particularly enjoyed the illustrated story because they identified with the Banda family, with 44% reporting that the story was the primary factor in motivating them to undertake the journey to QECH. Caregivers expressed a desire to care for their child as the Banda family did. Identification with the family seemed to ignite their hope and desire to go to QECH. Observing the Banda family’s experience of engaging with the health system gave several caregivers the impression that their child’s hearing would be restored if they attended QECH. One said:

*The story that I liked in the booklet is: the parents followed the counselling and their child’s hearing was restored. And the child was happy when he started hearing.*

*[Caregiver 315]*

While the story appeared to ignite hope, it may also have led families to have unrealistic expectations about the outcome of the referral, as well as masking some of the complexities faced in taking up the referral in reality. This may have been demotivating subsequently when the complex process of uptake posed greater challenges than expected. However, counselling with the booklet succeeded in setting the scene to imagine inclusion in the health system, piquing curiosity, prompting questions and generating hope. 

##### Providing Instructions

Many respondents expressed appreciation about the map included in the booklet, and 81% of those who went to QECH used it to find the ENT department. The detailed information about the journey, including the map, cost of transport and the photographs of the doctors in the ENT and audiology departments, was widely valued. When one of the caregivers showed the booklet to her family at home, she said:

*They took it and looked at it and said that it was good that the booklet provided directions. It will be easy to travel because it showed where we were going and the doctors who will greet us.*

*[Caregiver 105]*

##### Sharing the Booklet with Networks

The booklet was also used to convey the information and motivation generated at the camp with others. Discussing the referral with family, close friends and neighbours helped caregivers obtain crucial social support. Indeed, all bar one of the caregivers who attended the camp shared the booklet with the child’s other caregivers, and some explained how that helped them share knowledge and receive encouragement to attend. Describing this experience, one said:

*Their views about the booklet were that this is a good example, and they felt that this was a good time [to prioritise the problem]; maybe God was answering [our prayers] in that way and we should source money so that the child could go.*

*[Caregiver 308]*

The only caregiver who did not read the booklet (due to poor eyesight) still managed to mobilise crucial support, in part thanks to the reputation the booklet gained in the community, and the way the booklet was used to initiate and support conversations about the referral and the associated costs among their support networks.

*I was refusing [uptake] since I didn’t have money for transport; if am not able to find soap in my house, and salt too; and to find 10 thousand kwacha for transport for two people, I wouldn’t make it. And that’s when another woman [neighbour] said “can you bring your booklet and let me look at it”. And they read the [referral] letter and they told me that no matter what, I should do what I can to find money for transport and go with the child.*

*[Caregiver 104]*

### 3.3. Counselling and Booklet: Acceptability

#### 3.3.1. Time Taken 

In terms of feasibility, the main drawback of personal counselling was that it was time consuming. Counsellors reported that a few people complained about waiting, although none of the caregivers mentioned it in the interviews. One of the counsellors claimed that at least one caregiver had left the camp after a long wait because she was fed up with waiting. To reduce waiting times in the future, counsellors suggested training more counsellors for outreach camps. 

Including the counselling in camps also had its limitations. First, one counselling session could not address all the caregivers’ concerns, primarily because the topic was so unfamiliar that it took some time for the information to be absorbed. Although the caregivers said they were satisfied with the counselling sessions, 76% of them also reported wanting more information after the camp. Secondly, in 13% of cases, it was not the primary caregiver who attended the camps, and therefore these primary caregivers missed the opportunity to speak directly with counsellors. 

#### 3.3.2. Comprehension 

Text-based information and education were perceived as helpful; most of the sample (83%) were literate and those who could not read tended to ask for help and receive it. A counsellor explained:

*The good thing is that there are people who can read and others who cannot. Nowadays those who cannot read are few and if there is someone who can’t read, they always ask what is written. That means someone will read for them and they can keep the message that they have been told.*

*[Expert mother]*

However, the interviews exposed a gap between the ability to read and levels of reading comprehension, suggesting that the booklet primarily functioned as a pedagogical tool for counsellors. Almost half (47%) of the caregivers only had primary education, and without counsellor guidance, many caregivers said that they, or others, would have struggled to engage with all the content of the booklet and several had difficulty remembering the content. Evidently, counsellors used the booklet in such a way that it was accessible, because caregivers did not have to summon the motivation to read the booklet or find someone to help them read it.

#### 3.3.3. Scale-Up

Implementers involved in the intervention delivery appreciated and wanted to further develop the intervention. They suggested incorporating the booklet and counselling into local health centres to increase familiarity with QECH more broadly within Thyolo communities. For instance, a counsellor emphasised the value of the booklet in educating the community:

*More HSAs should be trained so that this message can reach the villages. If the people get the message in their homes and villages, maybe they can do something about it. Most people do not know that an ear problem is a problem [that can be treated]. They just keep the children at home. But if they receive the message from us, some people would go on their own to QECH because the booklet has a map. It explains the directions very well. And it also explains the money—how much you will spend - so the person can understand, even be enlightened, about money. One can decide on their own that “this child of mine, I should do this to take her to the hospital, I should not wait for the camp”.*

*[Counsellor]*

### 3.4. Text Message Reminder: Acceptability 

#### Prompt for Caregivers to Take Action 

The text messages served as additional prompts for caregivers to set in motion their preparations for the trip, primarily by raising funds for transport to the hospital. Confirmation of appointment dates provided clear fundraising deadlines. Most caregivers said they found the message to be a useful reminder; one caregiver explained that although the text message helped her make the decision to attend, she only made the journey after receiving a second reminder, which reminded her that help was available. Another explained how it made him feel cared for:

*This message is very good and can help a lot of people and be an encouragement to say, ‘oh the trip is on and the hospital is reminding us that we have this problem’. …reminding you that you have a problem is very helpful because it seems that you have someone who cares for your problem.*

*[Caregiver 108]*

However, the cue for action also provoked anxiety in some primary caregivers about their ability to raise the necessary funds in time. For the most part, feelings of anxiety about the responsibility caregivers faced seemed to be offset by appreciative feelings for the support they were receiving. The text message was encouraging for the caregivers who believed they could raise the money because it reminded them of the support available: the hospital cared about their individual case, the referral appointment was official, and they had been given a place in a broader health system.

The positive, respectful, welcoming tone of the text message and the number to call for questions were both considered helpful. Some used the phone number to postpone the appointment in order to gain additional time to raise funds for transport. However, others were unclear about the possibility of rescheduling appointments when they received the text message. One caregiver, who said she did not go because of lack of transport, implied a need to be told about flexible appointments:

*If the person who brought the [text] message told me that if you fail [to raise the money in time] you can also call and tell them that we’ve failed because of these reasons, then I would have tried to call, but they didn’t say that.*

*[Caregiver 310]*

In sum, the message was most acceptable to caregivers who were ready to attend the referral. For those who were not yet ready, they helped keep caregivers focused on their goal.

### 3.5. Text Message Reminder: Feasibility 

#### Network and Phone Coverage

Mobile phones as a medium for reminders were not as feasible as expected. Although the majority of caregivers (83%) had some kind of access to mobile phones, limited electricity supply and faulty networks meant that less than two-thirds (63%) of the caregivers received the text message. This prompted many interviewees to suggest using radio broadcasts, rather than text messages, to remind and prompt caregivers to take their children to hospital. Others suggested involving village headmen in delivering the message. One caregiver explains why radio would be useful:

*Yes, they should explain the messages concerning ears since more people at this time they listen to the radios. I think in that way the government can take part and tell people about ears, more people may know and would love to go to the hospital. Since there are other people who [have] the problem but don’t know where to go with it.*

*[Caregiver108]*

### 3.6. Residual Barriers to Uptake of Referral 

Although there were largely positive reflections on the intervention from caregivers, some were still unable to attend, suggesting that residual barriers exist which were not addressed by this intervention. The main residual barriers were the costs of seeking care and lack of exposure to hospitals and the city (discussed below). 

#### 3.6.1. Costs of Seeking Care

Accessing specialist healthcare incurs significant extra costs; the long journey to the hospital in Blantyre requires substantial funds for transport and food. Obtaining these funds is challenging in the context of an economy based on subsistence farming. None of the caregivers were able to afford transport to QECH on their own, so the cost of transport had to be shouldered communally by collecting donations from relatives and friends. This presented a range of challenges for primary caregivers, depending on their position within the family and community, and, for some, was not possible. As a caregiver who did not attend said:

*I was thinking that I will [ask] people in Johannesburg to send me money, but when I called them to tell them that there is this problem, they told me that they hadn’t been paid and it was too difficult. Now that I’m not working, I’m worried [we won’t be able to go].*

*[Caregiver 308]*

In this case, it was evident that motivation was not a barrier after the intervention; the caregivers took responsibility to try to raise the necessary funds to travel to hospital. However, for some, lack of financial resources remained an insurmountable barrier. One caregiver highlighted how a family emergency had eclipsed the importance of the referral, and used up the money set aside for it:

*There was no problem. Whatever happened here, everyone at home accepted it, and there was nothing to make us fail. We were just waiting. That’s when the sickness [of another family member] came. So when the time [for the child’s appointment] came close, we had spent the money [we had collected] for transport.*

*[Caregiver 101]*

Transport provision was considered unfeasible at the participatory workshop at which the intervention was designed [23]. However, in this study, both local health workers and caregivers believed it could be possible. 

#### 3.6.2. Lack of Exposure to Hospitals and the City

Lack of experience travelling to QECH reflected a broader inexperience and unfamiliarity with the culture of hospitals and biomedicine, which inevitably raised some fear in a small number of caregivers, despite the intervention’s efforts to address this. Fears ranged from financial issues, such as being caught in Blantyre longer than expected without accommodation or money for food, to fears that the child’s ear would be cut off in surgery. 

### 3.7. Intervention Costs

The cost per person for the whole intervention package, including development (designers, focus groups, training) costs, was £110. Considering the delivery costs only (printing, salaries, transport for counsellors), the cost per camp was £3.7.

## 4. Discussion

### 4.1. Acceptability and Feasibility of the Intervention

This study examined the acceptability and feasibility of an educational booklet, delivered with tailored individual counselling, and a text message reminder to address poor uptake of referrals for ear and hearing services. Testing the intervention in a small number of people from the target population highlighted the importance of pilot and feasibility testing, as outlined by the MRC guidance, in determining whether the intervention is acceptable, and which components of the intervention may need to be adapted prior to a wider trial. There are few examples of how the MRC guidance has been used to develop and evaluate interventions, particularly in LMICs. This study provides a useful case study for the process.

In terms of acceptability, the counselling enabled a two-way conversation about the referral and helped to dispel misconceptions and fear. The booklet provided motivation to attend, particularly through the illustrated storyline, and instructions on how to attend, and allowed information about the referral to be shared with social networks. Overall, this study suggested that individual counselling by an expert mother and HSA was an important factor in motivating caregivers to take up the referral. Primary caregivers could not afford to take up the referral on their own, but they all took responsibility to try to mobilise such support from others. The text message reminder was also found to be a valued prompt for caregivers. However, costs of transport to the hospital and competing priorities remained prohibitive for some families.

In terms of feasibility, a challenge from the perspective of the intervention implementers was the time taken for counselling. One alternative would be to deliver counselling in groups. A potential limitation of this is that counselling would less tailored to individual needs and uncertainties. However, there is some evidence that group-based peer support can allow parents to learn from the experiences of others, generate a shared social identity and improve health outcomes [24,25]. Thus, this approach deserves attention in further research.

Previous research has suggested promising findings regarding the use of text message reminders in improving access to health services in LMICs [12,13]. In our study, text message reminders provided a useful prompt to raise the necessary funds to take up the referral for those that received the message. However, given variable mobile phone and network coverage, questions remain as to the feasibility of this component in this setting. This may improve over time, with increasing network coverage and mobile phone ownership. In the meantime, alternative delivery mechanisms could be investigated, such as reminders to community health workers. This approach has been trialled in Kenya and Malawi to improve adherence to guidelines, with variable findings [26,27]. 

It is important that all individuals involved in caring for a child, including those who provide financial support (usually a male caregiver), understand the potential benefits of uptake. The ability to share the booklet helped in this regard. However, several caregivers recommended that local headmen could also bring attention to the social value of hospital referrals, given their respected positions in the community. This idea has been supported by research in Tanzania which found that training village leaders or schoolteachers to deliver health education about trichiasis surgery improved uptake [28]. Radio programmes were also suggested by implementers in this study. While there is some evidence of a positive impact of radio and other mass media on child survival health behaviours (participation in immunisation campaigns or bednet use), evidence on impact on referral uptake for specialist health services is lacking [29]. 

### 4.2. Overcoming Barriers

In contrast to the earlier formative research, where uptake of referral at outreach camps was only 3%, uptake following the intervention was 53% [11], although direct statistical comparisons are not possible, given that these included different study populations and time points. The reasons for poor uptake in the previous study were identified to be [14]:

- Location of the hospital,
- Lack of and cost of transport,
- Other indirect costs of seeking care,
- Fear and uncertainty about the referral hospital,
- Lack of understanding about the referral,
- Lack of awareness and understanding of hearing loss,
- Lack of visibility and availability of services.

Comparing these barriers to the findings of the current study, fear or uncertainty about attending QECH was rarely expressed. Furthermore, there was good understanding about the referral process; caregivers knew when and where to attend, and had good understanding about their child’s condition and need for treatment. Overall, the findings suggest the intervention may have helped to overcome some of the key barriers previously identified. 

However, the study found that cost of transport remains an important barrier to uptake, and that no amount of motivation can overcome material scarcity. Transport is widely recognised as an issue in research around barriers to accessing health care [30,31,32]. Key stakeholders in our intervention development workshop (including health workers, NGO staff and caregivers) did not prioritise transport provision due to concerns relating to sustainability [23]. However, there is some evidence—mostly for emergency maternal and child health—that transportation provision is an effective mechanism to support access to healthcare, in addition to other activities such as community education and healthcare facility improvements [33]. Community loan programmes or fuel vouchers have also been found to overcome transportation costs and improve access to healthcare [33]. In light of our finding that most caregivers borrowed money or used savings to attend the referral, and that those who did not attend were unable to borrow money from their networks, a formalised community loan programme, for example a village savings loans association, may be beneficial and deserves further attention in this setting [34]. 

Thyolo is very underserved in terms of ear and hearing health, with only one ENT clinical officer based at the district hospital. While outreach programmes help to improve access in the short term, long-term solutions aimed at increasing the availability of services in rural areas are also necessary. Recent work in Malawi has shown that training community health workers in ear and hearing care is feasible, which has the potential to improve access [35].

### 4.3. Adaptations Prior to Randomised Trial 

In summary, all intervention components were acceptable to and valued by participants. The feasibility of the text message reminders may have been limited by network coverage. As an alternative, when rolling out the intervention to a wider trial, the text message reminders could be sent to community health workers. The time taken to deliver individual counselling may also limit the feasibility of delivery. Time may be reduced by delivering counselling in groups. Involvement of community leaders in the intervention to improve uptake of referral may also be valuable. In addition, as residual financial barriers exist for many caregivers, an additional intervention component addressing these barriers should be included. This may be vouchers for transportation or a community loan programme, which have shown promising results in other settings. These adaptations will be explored before a full randomised trial is implemented. This will likely involve stakeholder engagement and small-scale piloting of the adapted intervention with the target population.

### 4.4. Strengths and Limitations

This research explored acceptability and feasibility in depth, which is a key step in the process of developing and evaluating complex interventions [14]. A mixed methods approach was used, and both caregivers and implementers were interviewed, enabling exploration of different perspectives and triangulation of findings. Data were collected at baseline and follow-up, which allowed us to explore acceptability and feasibility in different behaviour settings (the camp and at home), and we conducted an in-depth anthropological analysis of the data. The intervention targeted caregivers of children with hearing impairments, however, adaptations could make it applicable to a wider population group, for example, children with other impairment types. 

This study has some limitations that need to be taken into account when interpreting the results. Firstly, the small sample size and lack of comparison group (without the intervention) in this study meant that we were unable to assess effectiveness of the intervention; this would require a randomised controlled trial. The small sample size means that the experiences reported may not be generalisable to all caregivers of children with hearing loss in the Thyolo district. Secondly, we were unable to examine sub-population differences, i.e., whether uptake differed by type of referral. Finally, two of the outreach camps where the intervention was delivered were relatively small; it is possible that delivery of the intervention with one-to-one counselling is more feasible in the smaller camps compared to busier camps, and this needs further testing. 

## 5. Conclusions

Addressing poor uptake of referral from outreach camps is crucial for improving the ear and hearing health of children in rural areas of Malawi. This study provides an example of a feasibility study of an intervention to improve uptake—a process recommended by the MRC as a key component of intervention development prior to embarking on a full randomised controlled trial. We found that a fairly simple, low-cost, multi-component intervention was generally acceptable and feasible, and helped to overcome some key barriers to uptake. Counselling with an information booklet helped caregivers visualise uptake, ask further questions and understand the logistical requirements for uptake. The illustrated story piqued curiosity and inspired engagement, and counselling helped addressed caregivers’ fears. The text message reminder was found to be a valued prompt for caregivers. However, there were also challenges, including the time needed to implement the intervention, residual financial barriers for some families and low network and phone coverage. These findings have highlighted how the intervention can be to adapted and improved in order to maximise impact in a trial. 

## Figures and Tables

**Table 1:** Characteristics of the sample.

Child Characteristics	*n* (%)
Mean age (range) (years)	10.5 (4–16)
Sex	
Male	13 (43%)
Female	17 (57%)
Duration of hearing difficulty	
<1 year	7 (23%)
Between 1–5 years	18 (60%)
>5 years	4 (13%)
Don’t know	1 (3%)
Attend school (among those of school age)	27 (100%)
Grade	
Same year as other children	10 (37%)
Lower grade than other children their age	16 (59%)
Higher grade than other children their age	1 (3%)
Referral type	
Hearing assessment/hearing aids	11 (37%)
Surgery	19 (63%)
Caregiver characteristics **	
Sex	
Male	6 (20%)
Female	24 (80%)
Age group (years)	
19–29	9 (30%)
30–39	14 (47%)
40–49	7 (23%)
Mean age (range) (years)	34.0 (19–49)
Primary caregiver	
Yes	26 (87%)
No	4 (13%)
Relationship to child	
Mother	19 (63%)
Father	5 (17%)
Grandparent	1 (3%)
Other	5 (17%)
Father lives in same household	
Yes	16 (53%)
No	14 (47%)
Father contact in last 6 months	
Daily	16 (53%)
Monthly	3 (10%)
Once every 6 months	2 (7%)
Less often	1 (3%)
Father not alive	6 (20%)
Unknown	2 (7%)
Literacy	
Literate	25 (83%)
Illiterate	5 (17%)
Currently Working	
Yes, full time	8 (26%)
Part time	4 (13%)
No	18 (60%)

** for two caregivers, two children were referred (i.e., total number of caregivers was 28).

**Figure A1:**
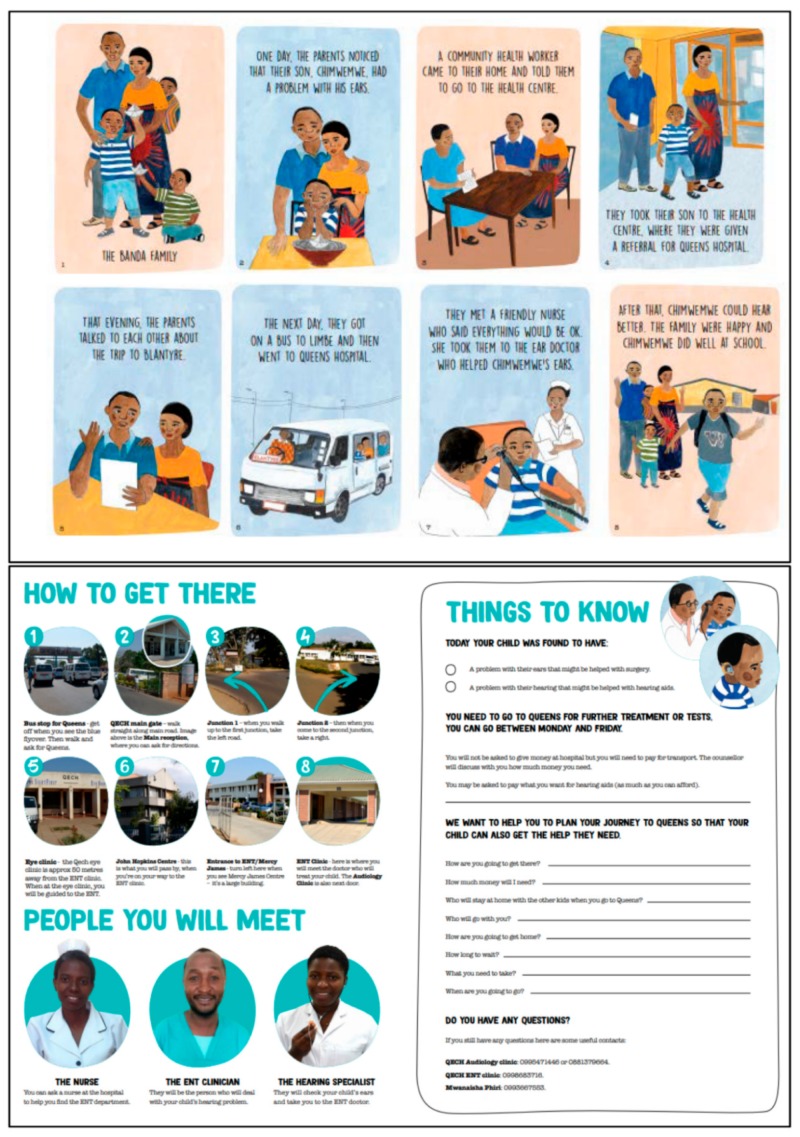
Booklet.

## References

[1] World Health Organization Hear the Future 2018. http://www.who.int/deafness/world-hearing-day/World-Hearing-Day-Infographic-EN.pdf?ua=1.

[2] Tesni S. (2014). Going to school with a hearing impairment. Ear Hear. J..

[3] Woodcock K., Pole J.D. (2008). Educational attainment, labour force status and injury: A comparison of Canadians with and without deafness and hearing loss. Int. J. Rehabil. Res..

[4] Asghari A., Farhadi M., Daneshi A., Khabazkhoob M., Mohazzab-Torabi S., Jalessi M., Emamjomeh H. (2017). The Prevalence of Hearing Impairment by Age and Gender in a Population-based Study. Iran. J. Public Health.

[5] Olusanya B.O., Neumann K.J., Saunders J.E. (2014). The global burden of disabling hearing impairment: A call to action. Bull. World Health Organ..

[6] Banks L.M., Kuper H., Polack S. (2017). Poverty and disability in low- and middle-income countries: A systematic review. PLoS ONE.

[7] Skilton M.K., Poole N., Metcalfe C.W., Martin T.P.C., Smith M.C.F. (2016). The impact of ear disease and hearing impairment on the lives of Nepali patients in Pokhara: A qualitative study. Int. Health.

[8] World Health Organization Childhood Hearing Loss: Strategies for Prevention and Care 2015. http://apps.who.int/iris/bitstream/handle/10665/204632/9789241510325_eng.pdf?sequence=1.

[9] World Health Organization (2013). Multi-Country Assessment of National Capacity to Provide Hearing Care. http://www.who.int/pbd/publications/WHOReportHearingCare_Englishweb.pdf.

[10] Mulwafu W., Ensink R., Kuper H., Fagan J. (2017). Survey of ENT services in sub-Saharan Africa: Little progress between 2009 and 2015. Global Health Action.

[11] Bright T., Mulwafu W., Thindwa R., Zuurmond M., Polack S. (2017). Reasons for low uptake of referrals to ear and hearing services for children in Malawi. PLoS ONE.

[12] Bright T., Felix L., Kuper H., Polack S. (2017). A systematic review of strategies to increase access to health services among children in low and middle income countries. BMC Health Serv. Res..

[13] Bright T., Felix L., Kuper H., Polack S. (2018). Systematic review of strategies to increase access to health services among children over five in low- and middle-income countries. Trop. Med. Int. Health.

[14] Medical Research Council Developing and Evaluating Complex Interventions: New Guidance 2006. https://mrc.ukri.org/documents/pdf/complex-interventions-guidance/.

[15] Eldridge S.M., Lancaster G.A., Campbell M.J., Thabane L., Hopewell S., Coleman C.L., Bond C.M. (2016). Defining Feasibility and Pilot Studies in Preparation for Randomised Controlled Trials: Development of a Conceptual Framework. PLoS ONE.

[16] Ásbjörnsdóttir K.H., Ajjampur S.S.R., Anderson R.M., Bailey R., Gardiner I., Halliday K.E., Ibikounle M., Kalua K., Kang G., Littlewood D.T.J. (2018). Assessing the feasibility of interrupting the transmission of soil-transmitted helminths through mass drug administration: The DeWorm3 cluster randomized trial protocol. PLoS Negl. Trop. Dis..

[17] Lewycka S., Mwansambo C., Kazembe P., Phiri T., Mganga A., Rosato M., Chapota H., Malamba F., Vergnano S., Newell M.-L. (2010). A cluster randomised controlled trial of the community effectiveness of two interventions in rural Malawi to improve health care and to reduce maternal, newborn and infant mortality. Trials.

[18] Arain M., Campbell M.J., Cooper C.L., Lancaster G.A. (2010). What is a pilot or feasibility study? A review of current practice and editorial policy. BMC Med. Res. Methodol..

[19] Smith J.A. (2011). Evaluating the contribution of interpretative phenomenological analysis. Health Psychol. Rev..

[20] Smith J.A., Osborn M. (1999). Doing Interpretative Phenomenological Analysis. Qualitative Health Psychology: Theories and Methods.

[21] Braun V., Clarke V. (2006). Using thematic analysis in psychology. Qual. Res. Psychol..

[22] Allen M. (2017). The SAGE Encyclopedia of Communication Research Methods.

[23] Wilbur J., Bright T., Mahon T., Hameed S., Torondel B., Mulwafu W., Kuper H., Polack S. (2018). Developing Behaviour Change Interventions for Improving Access to Health and Hygiene for People with Disabilities: Two Case Studies from Nepal and Malawi. Int. J. Environ. Res. Public Health.

[24] Shilling V., Morris C., Thompson-Coon J., Ukoumunne O., Rogers M., Logan S. (2013). Peer support for parents of children with chronic disabling conditions: A systematic review of quantitative and qualitative studies. Develop. Med. Child Neurol..

[25] Peers for Progress (2014). Global Evidence for Peer Support: Humanizing Health Care 2014. Peers for Progress.

[26] Kaunda-Khangamwa B.N., Steinhardt L.C., Rowe A.K., Gumbo A., Moyo D., Nsona H., Troell P., Zurovac D., Mathanga D. (2018). The effect of mobile phone text message reminders on health workers’ adherence to case management guidelines for malaria and other diseases in Malawi: Lessons from qualitative data from a cluster-randomized trial. Malar. J..

[27] Zurovac D., Sudoi R.K., Akhwale W.S., Ndiritu M., Hamer D.H., Rowe A.K., Snow R.W. (2011). The effect of mobile phone text-message reminders on Kenyan health workers’ adherence to malaria treatment guidelines: A cluster randomised trial. Lancet.

[28] Mahande M., Tharaney M., Kirumbi E., Ngirawamungu E., Geneau R., Tapert L., Courtright P. (2007). Uptake of trichiasis surgical services in Tanzania through two village-based approaches. Br. J. Ophthalmol..

[29] Naugle D.A., Hornik R.C. (2014). Systematic review of the effectiveness of mass media interventions for child survival in low- and middle-income countries. J. Health Commun..

[30] Jacobs B., Ir P., Bigdeli M., Annear P.L., Van Damme W. (2012). Addressing access barriers to health services: An analytical framework for selecting appropriate interventions in low-income Asian countries. Health Policy Plan..

[31] Kazibwe H., Struthers P. (2009). Barriers experienced by parents of children with clubfoot deformity attending specialised clinics in Uganda. Trop. Doct..

[32] Lee B.W., Sathyan P., John R.K., Singh K., Robin A.L. (2008). Predictors of and barriers associated with poor follow-up in patients with glaucoma in South India. Arch. Ophthalmol..

[33] Raje F. Rural Transport Interventions to Improve Maternal Health Outcomes 2018. https://assets.publishing.service.gov.uk/media/5b69d458e5274a1521d88e95/Maternal_health_and_transport.pdf.

[34] Ksoll C., Lilleør H.B., Lønborg J.H., Rasmussen O.D. (2016). Impact of Village Savings and Loan Associations: Evidence from a cluster randomized trial. J. Develop. Econ..

[35] Mulwafu W., Kuper H., Viste A., Goplen F.K. (2017). Feasibility and acceptability of training community health workers in ear and hearing care in Malawi: A cluster randomised controlled trial. BMJ Open.

